# SARS-CoV-2 Infection, A Risk Factor for Pituitary Apoplexy? A Case Series and Literature Review

**DOI:** 10.1177/01455613231179714

**Published:** 2023-06-08

**Authors:** Christina Hazzi, Noémie Villemure-Poliquin, Sylvie Nadeau, Pierre-Olivier Champagne

**Affiliations:** 1 Department of Ophthalmology and Otolaryngology—Head and Neck Surgery, CHU de Québec, Quebec, QC, Canada; 2 Department of Neurosurgery, CHU de Québec, Quebec, QC, Canada

**Keywords:** COVID-19, endoscopic sinus surgery, panhypopituitarism, pituitary apoplexy, skull base surgery

## Abstract

**Introduction:** Pituitary apoplexy (PA) is a rare phenomenon, characterized by a hemorrhagic or ischemic event of the pituitary gland, most often in association with a pituitary lesion. Severe acute respiratory syndrome coronavirus-2 (SARS-CoV-2) is the strain of virus responsible for the internationally recognized global pandemic COVID-19. Multiple clinical manifestations associated with this virus have been described, ranging from asymptomatic, mild flu symptoms to acute respiratory distress syndrome, end-organ failure leading to death. Cases of patients with concomitant COVID-19 infections and PA are being further recognized in the literature, but the causal association between the 2 entities remains speculative. **Objectives:** The objectives of this case series are 3-fold: to describe additional cases of patients with concomitant COVID-19 infection and PA (1), to review the current evidence regarding this potential complication associated with a COVID-19 infection (2), and to discuss physiopathological hypotheses, treatments, and prognoses of this newly recognized association (3). **Method:** We conducted an electronic chart review of patients treated for PA with concomitant COVID-19 infection from March 2020 to December 2021. A literature review was performed using MEDLINE, Web of Science, and Embase databases to identify other cases of COVID-19-associated PA. **Results:** From March 2020 to December 2021, 3 patients presented to our center with PA following a symptomatic COVID-19 infection. Two of these patients developed PA symptoms days following the viral infection, whereas the third patient developed PA after a 2-month period. The 2 first patients were managed surgically because of persistent visual symptoms. Results from our literature review yielded 12 other cases of COVID-19-associated PAs. **Conclusions:** The association between COVID-19 infection and PA has been increasingly reported in the literature. With the addition of the 3 cases described in our article, a total of 15 cases have been published. Many contributing mechanisms may lead to PA following COVID-19 infection. Coagulopathy is probable major contributing cause responsible for hemorrhage or infarction of the pituitary gland. Our case series provides further arguments that PA may be a direct manifestation of a COVID-19 infection.

## Introduction

Pituitary apoplexy (PA) is a rare phenomenon, characterized by a hemorrhagic or an ischemic event of the pituitary gland, most often caused by a macroadenoma.^1^ Its incidence is difficult to evaluate and varies between 1% and 26%^1^ across the literature. The pathophysiology of PA is largely misunderstood and multiple hypotheses have been proposed thus far. Vascular compromise by outgrowth and compression by a macroadenoma are the accepted premises but fail to explain all cases of PA. However, the theory of expansion illustrates the clinical manifestations of the disease. The most frequently encountered symptoms being severe acute retro-orbital headache, nausea, visual impairment, and hypopituitarism.^2^ Diagnosis is based on computed tomography (CT) scan or magnetic resonance imaging (MRI) findings. Management of PA is either conservative or surgical. Evidence of severe disease with neurological deficits, visual field defects, and altered mental state^3^ generally require surgical management, but there is no consensus on criteria that warrant surgical versus conservative management.

Severe acute respiratory syndrome coronavirus-2 (SARS-CoV-2) is the strain of virus responsible for the internationally recognized global pandemic COVID-19. As of December 2021, the cap of over 260 million cases had been breached and over 5 million people have succumbed to the disease worldwide.^3^ There are multiple clinical manifestations associated with this virus, ranging from asymptomatic, mild flu symptoms to acute respiratory distress syndrome, end-organ failure and death.^4^ Vascular complications associated with the SARS-CoV-2, such as PA, are being further recognized in the literature.^5^

We describe a case series of 3 patients presenting with PA and a concomitant infection to SARS-CoV-2 virus strain.

## Case 1

**Figure 1.**
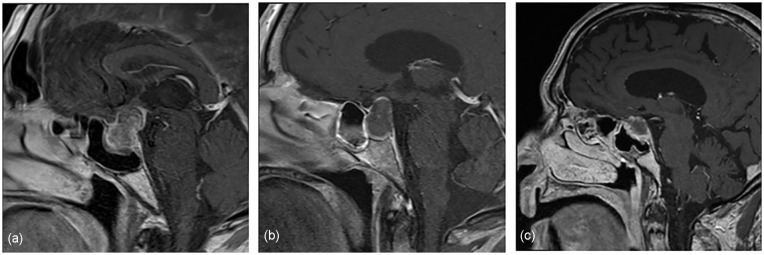
Contrast-enhanced T1WI magnetic resonance images showing (a) irregular internal and peripheral enhancement of the pituitary gland, compatible with a macroadenoma with suprasellar extension, (b) an increase in cephalocaudal dimension of a known macroadenoma with peripheral hemorrhagic contents, and (c) a pituitary gland lesion with intralesional hemorrhagic signals. T1WI, T1-weighted image.

A 65-year-old healthy man, presented to the emergency room (ER) with a persistent headache and left eye ptosis accompanied with a cough. Initial laboratory results revealed hyponatremia as well as a positive COVID-19 polymerase chain reaction test. A CT scan demonstrated an image compatible with a recent hemorrhage within a pituitary macroadenoma, causing PA. Further endocrine work-up revealed hypothyroidism, central adrenal insufficiency, as well as syndrome of inappropriate antidiuretic hormone, hyponatremia, all compatible with panhypopituitarism. On the third day of hospitalization, he developed ophthalmoplegia secondary to cranial nerve III, IV, VI paresis and ipsilateral hemianopia. An MRI, which was delayed due to patient claustrophobia, confirmed the presence of a macroadenoma with signs of recent hemorrhage, causing compression of the optic chiasma (Figure 1a). Considering the patient’s neurological symptoms, the decision was taken to perform surgical decompression of the sella turcica by transsphenoidal approach. Owing to the expositional risk associated with the aerosol-generating procedure, the surgery was delayed until day 13 post-COVID diagnostic. The neurosurgical team, with the help of an otolaryngologist, gained access to the sella turcica by endonasal approach, drained the hemorrhagic contents, and excised the pituitary macroadenoma. Pathology reports confirmed the diagnosis of a necrotized adenoma of the pituitary gland. The patient was discharged on postoperative day 5. Six-month follow-up demonstrated an improvement of the right visual field and a stable MRI image compatible with postoperative anatomical changes.

## Case 2

A 61-year-old woman with a known, asymptomatic, pituitary macroadenoma, and an intellectual disability presented to our ER with fever and lethargy 6 days after testing positive for a COVID-19 infection. She was hospitalized and developed right oculomotor nerve paresis as well as central hypothyroidism and adrenal insufficiency. Both CT scan and MRI demonstrated an increase in size of her known macroadenoma with hemorrhagic contents (Figure 1b). PA was diagnosed. Due to her neurological stability, a conservative approach was favored, and surgery was delayed. She was discharged after a 6-day hospitalization. During her follow-up appointments, there was no improvement in her neurological symptoms; therefore, surgery was indicated. She underwent transsphenoidal macroadenoma resection 33 days after her COVID-19 diagnosis. Pathologic examination confirmed the presence of extended necrosis of a pituitary macroadenoma. The patient had an uncomplicated postoperative course and was discharged on postoperative day 7. At the 2 month follow-up visit, oculomotor nerve paresis had completely resolved. A control MRI performed 2 months after surgery demonstrated anticipated postoperative anatomical changes.

## Case 3

An 89-year-old man previously known for hypertension and dyslipidemia developed a severe headache for which he presented to our ER. The patient had contracted and survived a COVID-19 infection 2 months prior to these symptoms. As initial laboratory work-up and CT scan imaging were negative, the patient was discharged with pain medication for a headache of unknown etiology. He sought medical attention 3 days later for a persistent, intense headache. A repeat CT scan demonstrated a 10-mm-thick lesion of the suprasellar region with heterogeneous enhancement associated with a suspected pituitary macroadenoma. He was admitted to the hospital and evaluated by the endocrinology team, who diagnosed him with panhypopituitarism. Initial MRI demonstrated an increase in size of the pituitary gland and enhancement compatible with internal hemorrhage of the lesion (Figure 1c), diagnostic of PA. The patient was observed for several days but did not develop any neurological symptoms. A conservative, non-surgical approach was therefore preferred. He was discharged on day 5 of admission with substitute hormonotherapy and a follow-up with the endocrinologist. Two-month and 6-month follow-up MRI exams demonstrated a reduction in size of the hemorrhagic intrasellar lesion with an underlying pituitary adenoma.

## Discussion

The 3 cases described herein are part of a scarce number of new reports of patients suffering from PA and a concomitant SARS-CoV-2 infection. To our knowledge, 9 case reports and 1 case series of 3 patients, for a total of 12 patients, have previously been reported in the literature. Results from our review of the literature are summarized in Table 1.

**Table 1.** Reported cases of PA in the setting of a confirmed COVID-19 infection.

Authors	Date published	Patient description	Comorbidities	Signs and symptoms PA	COVID-19 symptoms	Delay between COVID-19 symptoms and PA diagnostic	Treatment	Delay of surgical treatment	Postoperative period
Liew S-Y et al^6^	16/07/2021	72 Y.O. (Year Old) man	Mallory Weis	Severe frontal headache, fever, drowsiness No neurological symptoms ↓ testosterone, cortisol, TSH/T4	Mild hypoxemia and desaturation (95%), no supplemental O_2_	6 wk delay	Intravenous (IV) hydrocortisone + thyroid hormones	NA	Hormonal therapy replacement 6-month follow-up (F/U) MRI
Solorio-Pineda et al^7^	25/09/2020	27 Y.O. man	None	Glasgow coma scale 11, left eye exotropia and myosis ↓ testosterone	Asthenia, fever, desaturation, respiratory distress	+ test during hospitalization 4 d delay between COVID-19 symptoms and PA symptoms	Pulmonary management (mechanical ventilation) Anticoagulants, dopamine agonist	NA	Death 12 h after hospital admission
Ghosh et al^8^	02/01/2021	44 Y.O. woman	None	Severe, sudden headache, vomiting, blurred vision (bitemporal hemianopsia) ↓ cortisol, ↓Na	Fever	+ test during hospitalization 6 d delay between COVID-19 symptoms and PA symptoms	IV dexamethasone Antibiotics Refusal of surgical tx	NA	Hormonal therapy replacement 2-month F/U: improvement of hemianopia
Kamel, W. A et al^9^	28/06/2021	55 Y.O man	Diabetes Hypertension Known pituitary adenoma (resection 11 y ago with recurrence)	Severe headache, left oculomotor palsy, dilated non-reactive pupil, vision loss	Fever, myalgia, cough	+ test during hospitalization 6 d delay between COVID-19 symptoms and PA symptoms	Urgent TSS with tumor excision (COVID-19 precautions, air purifiers, and limited operating room (OR) staff)	No delay	Improvement of ptosis and left eye vision 3 d post-op, progression of COVID-19 symptoms, intubation and death
Chan et al^10^	20/08/2020	28 Y.O woman	38 wk pregnant	Left eye blurriness, mild headache, dilated left pupil ↓ACTH, ↓ TSH/T4, ↓ FSH/LH	Ear pain, myalgia, chills, rhinorrhea	+ test during hospitalization 3 d delay between COVID-19 symptoms and PA symptoms	Dexamethasone Induced vaginal delivery TSS postpartum day 2 (COVID-19 precautions, air purifiers, and limited OR staff)	1 wk between diagnostic and TSS	Complete visual recovery 2 mo F/U: hypothyroidism and hypogonadism
Santos et al^11^	31/05/2020	47 Y.O man	None	Frontal headache, diplopia, left eye ptosis, ↓ left visual acuity ↓ cortisol	Myalgia	+ test during hospitalization 3 wk delay between COVID-19 symptoms and PA symptoms	Ibuprofen TSS	Urgent (day 5 after diagnostic)	Discharge postoperative day 4
Bordes et al^12^	12/02/2021	65 Y.O. woman	Hypertension Fibromyalgia	Headache, vomiting, photophobia, phonophobia	Malaise and cough	+ test during hospitalization 1 mo delay between COVID-19 symptoms and PA symptoms	Hydrocortisone	NA	Hormonal therapy replacement
Ramamurthy et al^13^	16/03/2021	46 Y.O man	None	Sudden vision loss (light perception) in both eyes	Fever		IV cortisone Antibiotics	NA	Improvement in vision Awaiting neurosurgery
LaRoy and McGuire^14^	23/02/2021	35 Y.O man	None	Headache, neck stiffness	Fever, anosmia, dysgeusia, ↑ sputum	+ test during hospitalization 4 d delay between COVID-19 symptoms and PA symptoms	Antibiotics	NA	
Martinez-Perez et al^15^	08/2021	54 Y.O woman	None	Headache, blurry vision	COVID contact, no sx except headache	+ test during hospitalization	Transcranial resection	Urgent	Hormonal therapy replacement Improvement of vision (residual right hemianopsia)
		56 Y.O man	Obesity Hypertension Hypothyroidism	Headache, diplopia Cranial nerve III, IV palsy, altered mental state	Chills, myalgia	+ test during hospitalization 10 d delay between COVID-19 symptoms and PA symptoms	TSS	Urgent	Hormonal therapy replacement 6-wk F/U: complete resolution of nerve palsy
		52 Y.O man	Obesity Hypertension	Peripheral vision loss, impotence, ↓ TSH/T4, ↓ FSH/LH, ↓IGF, ↓ cortisol		+ test during hospitalization 4 d delay between PA symptoms and COVID-19 symptoms	TSS	Urgent (day 3 after diagnostic)	Hormonal therapy replacement Complete reversal of visual symptoms
Hazzi et al^16^		65 Y.O man	None	Headache, left eye ptosis, cranial nerve III, IV, VI palsy, homonym hemianopsia Panhypopituitarism	Cough	+ test during hospitalization Concomitant symptoms of PA and COVID-19	TSS	Day 13 after diagnostic of PA and COVID-19	Hormonal therapy replacement 6-mo F/U: improvement in right visual field, nerve palsy resolution
		61 Y.O woman	Known macroadenoma Intellectual deficiency	Cranial nerve III palsy Hypothyroidism, adrenal insufficiency	Fever, lethargy	6 d delay between + COVID-19 test and PA symptoms	Initial conservative management TSS after non-resolving ocular symptoms	Day 33 after COVID-19 diagnostic	Hormonal therapy replacement 2-month F/U: complete visual resolution
		89 Y.O man	Hypertension Dyslipidemia	Headache, panhypopituitarism		2 mo delay between + COVID-19 test and symptoms of PA	IV hydrocortisone + thyroid hormones	NA	6-month F/U: hormonal therapy replacement

Information was obtained during our literature review performed using MEDLINE, Web of Science, and Embase databases to identify other cases of COVID-19-associated pituitary apoplexies.

Abbreviations: ACTH, adrenocorticotropic hormone; FSH/LH, follicle-stimulating hormone/luteinizing hormone; IGF, insulin-like growth factor; PA, pituitary apoplexy; sx, symptoms; TSH, thyroid-stimulating hormone; TSS, transsphenoidal surgery; tx, treatment.

PA was first described in 1950,^1^ in a case series describing 5 patients suffering from sudden hemorrhage of the pituitary gland. This clinical entity is defined as a rapid expansion of a normal or neoplastic pituitary gland by sudden hemorrhage or infarction. Its incidence is difficult to establish and varies in the literature between 1% and 26%.^1^ Most cases of PA present in the fifth or sixth decade of life,^1^ as is reflected in 2 of our described patients. Two of our 3 patients were of male gender, mirroring the male predominance suggested in the literature.

Known risk factors of PA are hypertension, major surgery, dynamic testing of the pituitary gland, anticoagulation, dopamine receptor agonists, estrogen replacement therapy, radiation, pregnancy, and head trauma.^7,14^ Hypertension is in fact the most recognized precipitating factors of PA. It was also a frequently encountered comorbidity in previously published cases of patients with PA associated with COVID-19.^9,12,15^ PA is most often described with pituitary adenomas, but also can occur in glands suffering from hypophysitis, craniopharyngioma, Rathke cleft cysts, sellar tuberculoma, and sellar metastasis.^2^ In the cases described in this article, 1 patient had a known asymptomatic macroadenoma, whereas 2 patients presented were diagnosed initially with PA. In our review of the literature, 2 patients had the presence of a known macroadenoma^9^ while the others presented with PA as the first presenting sign.^6-8,10,11,13-15^ Only 1 case presented with PA in the absence of an underlying pituitary tumor.^12^

Adding our cases with the others that were already described, there are now 15 cases of patients who presented with PA after contracting a COVID-19 infection. This rising number of cases make it reasonable to believe that there may be a causal relationship between COVID-19 infection and PA. The temporality of this association is another argument for causality here. We found that 11 of the 15 patients described in the literature developed PA within 10 days of onset of COVID-19-related symptoms (fever, cough, dysgeusia, anosmia^17^), whereas 4 patients had up to 2 months of delay between COVID-19 diagnostic and neurological symptom presentation. In the cases described in this article, 1 patient presented with both symptoms and COVID-19, 1 patient developed PA a week following a positive COVID-19 test, and 1 patient developed PA 2-months following COVID-19 diagnosis.

The pathophysiology of PA remains unclear but multiple hypotheses have been explored. The high energy demand and limited blood supply of underlying adenomas make it an anatomic location with a fragile equilibrium. Any alteration in the balance between tumor perfusion and tumor metabolism increases the risk or hemorrhage or infarction.^2^ An infection by COVID-19 could play a role in disrupting that equilibrium but that role remains entirely hypothetical. Some authors have attempted to determine the route by which the virus enters and affects the central nervous system (CNS) and endocrine system. Entry into the CNS by coronavirus has been described through hematogenous or retrograde neuronal route, demonstrating the neurotropic and neuroinvasive capabilities of this pathogen.^18^ These statements corroborate with the finding of SARS-CoV viral particles in patients’ cerebrospinal fluid.^19^ The role of angiotensin-converting enzyme-2 (ACE-2) receptors has also been explored as an explanation for multi-organ involvement of the viral infection. ACE-2 receptors are found in the heart, gastrointestinal system, the CNS as well as in the coagulation cascade constituents and could act as points of entry of the virus inside the cells.^20^ Li et al^21^ demonstrated that ACE-2 can be immunoprecipitated by the S1 domain of SARS CoV-2 protein and that it can promote viral replication, proving its role as a functional receptor for the pathogen. This virus strain is therefore capable of neuroinvasion by direct viral infection of the gland, mediated by ACE-2 receptors.^18^

Other mechanisms of interaction between the endocrine, neural systems, and SARS CoV-2 have been proposed. Activation of the hypothalamo-pituitary axis via inflammatory mediators as well as an immune-mediated glandular damage causing hypophysitis, secondary to antibody formation or cell-mediated damage^22^ are all plausible ways coronavirus infection could interrupt neural function and lead to PA. There has been evidence to show that the virus in question induces an intracranial cytokine storm syndrome^18^ and subsequent breakdown of the blood–brain barrier rather than direct neural invasion.

Coagulopathy is another major probable contributing factor in the pathophysiology of PA following COVID-19 infections. As increasing cases were diagnosed worldwide, it rapidly became clear that COVID-19 infections had the potential to disrupt the coagulative hemostasis of patients and cause major thrombophilic complications. Fan et al^23^ described the cases of 4 young patients presenting with catastrophic, large arterial thrombosis at a median of 78 days from seroconversion from a COVID-19 infection.^23^ Two patients manifested large vessel ischemic stroke, one presented with an acute ischemic limb due to arterial thrombosis, and another had myocardial infarction. An increase in factor VIII, D-dimer levels, von Willebrand factor antigen, and hyperfibrinogenemia in these patients were suggestive of a hypercoagulable state. The duration of inflammation as well as thrombotic derangements induced by this novel viral infection has yet to be determined. A proposed state of endothelial activation and dysfunction and low-grade inflammation in the convalescent phase of the illness could be responsible for delayed thrombotic presentation and could explain both acute and late cases of patients suffering of PA following a COVID-19 infection.^23^ A systematic review by Tomerak et al^24^ also concluded that a hypercoagulable state is a common feature in many of the coronaviruses. A dysregulated immune response was found in these patients, leading to cytokine storm, endothelial cell activation, capillary leakage, hypotension, and hypercoagulation. Thrombosis ensues via activation of the coagulation cascade combined with downregulation of anticoagulating factors.

Management of PA is also of great debate and both conservative and surgical treatments have been used in the management of PA. Of the 15 published cases, 8 patients were treated surgically. Decreased visual acuity or ocular nerve palsy was considered surgical indication in all cases. Five of these patients were urgently operated, with little or no delay between diagnostic and surgical decompression. In the cases presented in our series, 2 patients underwent non-urgent transsphenoidal surgery due to persistent ocular symptoms. Current evidence suggests that transsphenoidal optic chiasma decompression is the recommended surgery for patients with PA presenting with impaired consciousness, hypothalamic involvement, sudden onset amaurosis, and decreased visual acuity.^7,14,15^ In contrast, conservative management is recommended for patients with isolated oculomotor involvement, unimpaired level of consciousness, no visual impairment, and subclinical PA.^14,15^ More recent evidence has shown that conservative management leads to favorable visual and neurological outcomes.^1^ Beyond the risks inherent to the surgery, there may be additional surgical risks associated with COVID-19 infections, for both surgeons and patients. Zou et al^25^ analyzed viral loads of SARS-CoV-2 and determined that viral quantity was independent of symptom severity and that higher viral loads were detected in the nasal mucosa soon after symptom onset. Both intranasal and endoscopic skull base surgeries are therefore high-risk procedures in an infected patient, putting the surgical team at threat for viral infection.^26^ The surgical risks are not only limited to the operating room (OR) staff. Carrier et al^27^ demonstrated that COVID-19 patients undergoing surgery are at greater risk of pulmonary and thromboembolic complications as well as a 15.9% risk of mortality.

In the cases presented herein, delay in surgery was not associated with worse outcome. Both patients who were treated surgically had a complete recovery at the postoperative follow-up visit. The decision to delay surgery was mainly due to the risk of viral shedding from active infection as well as the lack of evidence concerning indications for surgery.^28^ This contrasts with other published cases in which the majority of patients underwent urgent surgical management of PA. Only 1 case was treated with delayed surgery: a 28-year-old pregnant woman presenting with PA and a COVID-19 infection, operated 7 days after PA diagnostic,^10^ the reason for the delay being to allow childbirth. This patient also experienced complete visual recovery. However, postoperative evolution is difficult to compare in this instance. Of the 15 patients described, 2 succumbed to the viral infection and 3 are lacking information concerning follow-up. It is worth mentioning that all patients treated with delayed surgical management had a favorable evolution. The role operative delay will play in future similar cases is yet to be determined.

Finally, the SARS-CoV-2 pandemic has brought its fair share of obstacles related to surgical safety of staff members, access to the OR, and delays in surgery. These issues have forced us to revisit the operative indications for many surgical pathologies. The Pituitary Society Guidance recommends surgical delay in patients with COVID-19, ideally until they no longer have symptoms and have a negative swab test result. If emergent surgery is necessary and cannot be differed, they suggest alternative transcranial approaches to avoid nasal mucosa disruption. The replacement of high-speed drilling to non-powered tools as well as the use of large suction tubes to aspire particulate matter is recommended, jointly with the use of personal protective equipment.^29^ These guidelines may be subject to change with the ongoing evolution of the COVID-19 pandemic.

## Conclusion

In conclusion, an association between COVID-19 infection and PA has been increasingly reported in the literature. With the addition of the 3 cases presented in our article, a total of 15 cases have been described. The possible relation between COVID-19 and PA is becoming more and more evident, but a causal relationship between the 2 entities remains largely speculative. Although PA is a rare condition, COVID-19 cases keep rising as new mutant viral strains develop. If this infection is in fact a risk factor for developing PA, it is important to understand the pathophysiological interaction of the 2 entities to recognize the clinical presentation, quickly introduce the appropriate treatment, and establish recommendations concerning management. Many contributing mechanisms may lead to PA following COVID-19 infection. Our case series explores numerous hypotheses which may explain how PA may be associated with COVID-19 infection through the multiple pathogenic mechanisms previously described. We believe that publication and sharing of these cases is paramount and will certainly contribute to care and treatment optimization in patients with COVID-19 and PA.
